# Supplementary material for: Effects of low dose computed tomography (LDCT) on lung cancer screening on incidence and mortality in regions with high tuberculosis prevalence: A systematic review
Source: PLoS One. 2024 Sep 11;19(9):e0308106. doi: 10.1371/journal.pone.0308106 (PMC11389911; doi:10.1371/journal.pone.0308106)
Supplement: Attachment — Submitted filename: PONE-D-22-22894-Answers to reviewers-12-07.docx [file pone.0308106.s002.docx]

Dear Editor-in-Chief of Plos One,

Please find below the comments and answers for the Protocol that was submitted to PLOS ONE under the number “PONE-D-22-22894”.

Reviewer #1: the project is interesting and methodologically well conducted but I does not think that the publication of only the protocol is relevant as so far as this type of work has already been carried out and recently published in developed countries. it would be more interesting to publish it with the results.

The systematic review was completed and the manuscript in its final version is being submitted.

Reviewer #2: The manuscript provides the protocol for a systematic review of the effect of low dose CT (LDCT) on lung cancer mortality with a focus on populations with high tuberculosis (TB) prevalence. The authors address an important public health issue and their methodologic approach is appropriate.

However, both the review question and the applied methodology should be refined and described in more detail.

The reviewer evaluated the systematic review protocol, the authors reviewed the protocol based on the comments, and the systematic review manuscript was written considering the protocol evaluation.

The applied methodology was adjusted to attend to the reviewer's comments.

The effect of LDCT screening on lung cancer mortality is well studied and its benefits are acknowledged. The authors cite relevant reviews in their introduction that evaluate this very question. The new aspect in their planned review is the focus on populations with high TB prevalence. The authors should stress this aspect more to make their study unique. The criteria for regions or populations with high TB incidence should be defined in advance.

The title of the systematic review was changed to highlight how the study is unique. The regions considered as having high incidence/prevalence of TB were defined in advance.

The authors make two key arguments why the focus on TB populations is relevant: 1. Because TB is a risk factor for lung cancer, and 2. because subjects with TB are more likely to have false-positive LDCT results. The authors should make clear their hypothesis regarding the association of these two factors and the outcomes, particularly lung cancer mortality. Also, the association between TB prevalence and the economic situation of a country should be considered. If TB is more prevalent in low income countries, this might confound the effect of LDCT screening on mortality.

The authors appreciate the comments. We considered the aspects of association between high incidence/prevalence of TB and economic situation was well as TB as a risk factor for lung cancer and its impact on of LDCT screening in the discussion section. It was not a hypothesis of this study.

The authors aim to include three types of studies: randomized clinical trials, clinical trials without control group and observational cohort studies. The large RCT come from Europe and the US, regions not known to have high TB incidence. It is possibile that not all study types might be available from TB regions.

The authors appreciate the comments. In the final methodology, the authors decided to include RCTs and cohort studies with the control group.

After evaluating 14,143 publications, 37 were included in this systematic review, pertaining to 22 different studies (10 RCT and 12 cohorts). Only one RCT was from a country with a high incidence/prevalence of TB.

Specific comments regarding the review protocol concern the following:

- Review methodology: it is a plus that the authors follow the standards of the Handbook of the Cochrane Collaboration.

- Risk of bias: in the abstract the authors state that the Cochrane risk of bias tool is applied to avoid the risk of bias. However, the tool is not intended to avoid bias in completed studies, but to assess the risk of bias in the included studies. How will the risk of bias of the non-RCT be assessed?

The manuscript was modified to include the reviewer’s comments. There is a Cochrane tool to evaluate the risk of bias for the non-RCT. The disagreements were resolved by discussion and consensus.

- Data extraction: data on technical equipment, CT reading, case definition and case management should be extracted as they affect false-positive rates and detection rates.

The Threshold of abnormal LN (case definition) was extracted. Nonetheless, data on equipment and CT reading were not included in the parameters.

- Data analysis: the Cochrane Handbook cautions against vote counting. The authors should explain, why they still decide to use this method.

The interventions were described using the Template for Intervention Description and Replication (TIDIeR) checklist. We used incidence rate ratios (IRRs) method to assess if there is any evidence of the effect of LDCT screening on the outcomes. This was done separately for cohort studies and Clinical Trials.

- Evidence synthesis: the authors should specify what statistics software they will use for meta-analysis. The authors should describe in more detail how they plan to combine the results (effect estimates) from the different study types.

The interventions were described using the Template for Intervention Description and Replication (TIDIeR) checklist. The authors decided to not perform a meta-analysis. The results from the systematic review were summarized.

- Language: linguistic revision is recommended

The manuscript was completely reviewed for language and grammar.

Reviewer #3: The authors ask an interesting question, however the protocol requires further work.

1. Title- please specify what kind of mortality, and also include specifics on population being evaluated as this review is targeted.

The title was modified.

2. The text is poorly written. There are areas that need to be reviewed in terms of spelling and grammar. E.g. pg 3 line 9 from bottom - Two big clinical trial, and line 1 from bottom- Nevertheless, here are evidence.

The manuscript was reviewed to address language and grammar.

3. Introduction

a. The authors have listed adoption of lung cancer screening as essential - how have they justified this and in which population?

The word “essential” was removed to attend to the reviewer comments.

b. The authors state there have been mortality reductions observed in the NLST and NELSON trials- they should be careful with such broad statements and clarify what type of mortality reduction and also clarify in which populations

The authors included which type of mortality, which is 5-year mortality.

c. The introduction provides clinical recommendations - I would question the appropriateness of this in a systematic review protocol.

The clinical recommendations were removed to attend to the reviewer comments.

4. Objectives

a. Can the authors please justify why they decided to include cohort studies in addition to RCTs?

The decision to include cohort studies was to broaden the scope of the systematic review, specially aiming to include countries/regions with high incidence/prevalence of TB.

5. Methods

a. Can the authors elaborate on which information will be collected during the review process?

We extracted the following information: Study, year, Study type, Comparison provided, Country, High TB incidence, Sample size (N), Participation rate (%), Mean age, Target age (years), % Male, Smoking history required, Pack-years; years since quitting	Screening rounds (N), Time between rounds (years), Follow-up (years) and Threshold of abnormal LN.

b. Can the authors provide justification about why they have elected to use the vote counting method?

We changed the method and did not use vote counting following the reviewers’ considerations.

c. Is there any required duration of follow up for mortality outcomes?

We did not include the duration of follow-up as an inclusion criterion, we extracted the follow-up information and reported accordingly.
