# Supplementary material for: Effects of low dose computed tomography (LDCT) on lung cancer screening on incidence and mortality in regions with high tuberculosis prevalence: A systematic review
Source: PLoS One. 2024 Sep 11;19(9):e0308106. doi: 10.1371/journal.pone.0308106 (PMC11389911; doi:10.1371/journal.pone.0308106)
Supplement: Attachment — Submitted filename: PONE-D23-38324- Response to Reviewer-03-31-2024.docx [file pone.0308106.s003.docx]

Dear Editor-in-Chief of Plos One,

Thank you for the meticulous review of the manuscript. Below are the comments and answers for the manuscript submitted to PLOS ONE under the number “PONE-D-23-38324”.

Kind Regards,

Isabel Emmerick

Reviewer’s Responses to Questions

Comments to the Author
1. Is the manuscript technically sound, and does the data support the conclusions?
The manuscript must describe a technically sound piece of scientific research with data that supports the conclusions. Experiments must have been conducted rigorously, with appropriate controls, replication, and sample sizes. The conclusions must be drawn appropriately based on the data presented.
Reviewer #1: Yes
Reviewer #2: Yes
________________________________
2. Has the statistical analysis been performed appropriately and rigorously?
Reviewer #1: Yes
Reviewer #2: N/A
________________________________
3. Have the authors made all data underlying the findings in their manuscript fully available?
The PLOS Data policy<[http://www.plosone.org/static/policies.action#sharing](https://nam12.safelinks.protection.outlook.com/?url=http%3A%2F%2Fwww.plosone.org%2Fstatic%2Fpolicies.action%23sharing&data=05%7C02%7Cisabel.emmerick%40umassmemorial.org%7C84e94f05cb50438b1dfc08dc310c1482%7C9910941497df4111a54a633909f39003%7C0%7C0%7C638439175329307295%7CUnknown%7CTWFpbGZsb3d8eyJWIjoiMC4wLjAwMDAiLCJQIjoiV2luMzIiLCJBTiI6Ik1haWwiLCJXVCI6Mn0%3D%7C0%7C%7C%7C&sdata=lnnfUOM0oE%2Bksu%2Bs8cLt402nKFEtgTfSk6Q2p9WXngM%3D&reserved=0)> requires authors to make all data underlying the findings described in their manuscript fully available without restriction, with rare exception (please refer to the Data Availability Statement in the manuscript PDF file). The data should be provided as part of the manuscript or its supporting information or deposited to a public repository. For example, in addition to summary statistics, the data points behind means, medians, and variance measures should be available. If there are restrictions on publicly sharing data—e.g. participant privacy or use of data from a third party—those must be specified.
Reviewer #1: No
Reviewer #2: Yes
Authors’ answers: All the data used to prepare the graphs and analysis are available in the body of the manuscript within the tables. Due to copyright restrictions, the original manuscripts can not be shared. Nonetheless, they are all appropriately cited in the paper and can be found online.

________________________________
4. Is the manuscript presented in an intelligible fashion and written in standard English?
PLOS ONE does not copyedit accepted manuscripts, so the language in submitted articles must be clear, correct, and unambiguous. Any typographical or grammatical errors should be corrected at revision, so please note any specific errors here.
Reviewer #1: Yes
Reviewer #2: No

Authors’ answers: An english review was perfomed
________________________________
5. Review Comments to the Author
Please use the space provided to explain your answers to the questions above. You may also include additional comments for the author, including concerns about dual publication, research ethics, or publication ethics. (Please upload your review as an attachment if it exceeds 20,000 characters)

Reviewer #1: Dear Author,
The manuscript presents a thorough investigation into the effects of low-dose computed tomography (LDCT) screening for lung cancer in regions with a high prevalence of tuberculosis (TB). The systematic review and meta-analysis approach are commendable and provide valuable insights into this important topic.
The methodology section is well-structured, and the use of the PICOS framework for defining eligibility criteria adds clarity to the research process. Adherence to PRISMA guidelines enhances the transparency and reproducibility of the study.
The statistical analysis appears to have been conducted appropriately, supporting the conclusions drawn by the authors. However, the lack of full availability of underlying data is noted, which may impact the reproducibility of the findings. It would be beneficial to address this limitation to strengthen the credibility of the research.
Overall, the manuscript is presented clearly and written in standard English, making it accessible to readers. The study contributes valuable insights into the effectiveness of LDCT screening in TB-endemic regions. Addressing the availability of underlying data would further enhance the quality and impact of the research.
Authors’ answers: Thank you for your comments. The purpose of meta-analysis is not to reanalyze the original data but to aggregate and synthesize the results reported in the studies included in the analysis. Meta-analysis provides a quantitative estimate or summary of the findings from multiple studies on a particular topic, allowing researchers to draw more robust conclusions than those based on individual studies alone. By pooling data from various sources, meta-analysis can identify patterns, trends, and overall effects that may not be apparent in single studies. Therefore, the focus is on presenting and interpreting the collective evidence. In addition, we systematically evaluate the quality of the papers included and the risk of bias. All summarized data is available within the manuscript tables, allowing the reproducibility of the findings.

Additionally, we included a phrase on not performing a re-analysis of the original data in the limitation section.

Reviewer #2: native English needs to be reviewed
small sample size needs to be widen the sample in order to fundamentalize the conclusions
It would be change some guidelines in the follow up and interpretation of cancers and TB
Authors’ answers: Thank you for your comments.

1) The English was revised prior to this version submission.
2) “small sample size needs to be widen the sample in order to fundamentalize the conclusions”

In our systematic review, we followed a specific protocol registered in the PROSPERO database (registry number CRD42022309581). We included all the available evidence that met the inclusion criteria and adhered to recommended quality international guidelines. Therefore, the term “sample size” is not applicable in systematic reviews, which are exhaustive by definition.

3) It would be change some guidelines in the follow up and interpretation of cancers and TB - About the guidelines in the usage of radiological investigations in TB and their narrow interpretation so to be following as much as possible the basic guidelines and present them in a clear manner.

We included the following text in the manuscript: “Insights from this literature review may inform future guidelines on delivering LDCT screening in low- and middle-income countries (LMICs) with high TB burden. Our results emphasize the importance of implementing structured screening programs.”
